# Identification and Interaction Analysis of Significant Genes and MicroRNAs in Pterygium

**DOI:** 10.1155/2019/2767512

**Published:** 2019-06-25

**Authors:** Siying He, Hui Sun, Yifang Huang, Shiqi Dong, Chen Qiao, Shuai Zhang, Chen Wang, Fang Zheng, Ming Yan, Guohua Yang

**Affiliations:** ^1^Center for Gene Diagnosis & Core Lab, Zhongnan Hospital of Wuhan University, Wuhan, Hubei 430071, China; ^2^Department of Ophthamology, Zhongnan Hospital of Wuhan University, Wuhan, Hubei 430071, China; ^3^Department of Corneal, Hankou Aier Eye Hospital, Wuhan, Hubei 430024, China; ^4^Hubei Clinical Research Center for Prenatal Diagnosis and Birth Health, Wuhan, Hubei 430071, China; ^5^Demonstration Center for Experimental Basic Medicine Education of Wuhan University, Wuhan, Hubei 430071, China

## Abstract

**Purpose:**

MiRNAs have been widely analyzed in the occurrence and development of many diseases, including pterygium. This study aimed to identify the key genes and miRNAs in pterygium and to explore the underlying molecular mechanisms.

**Methods:**

MiRNA expression was initially extracted and pooled by published literature. Microarray data about differentially expressed genes was downloaded from Gene Expression Omnibus (GEO) database and analyzed with the R programming language. Functional and pathway enrichment analyses were performed using the database for Annotation, Visualization and Integrated Discovery (DAVID). The protein-protein interaction network was constructed with the STRING database. The associations between chemicals, differentially expressed miRNAs, and differentially expressed genes were predicted using the online resource. All the networks were constructed using Cytoscape.

**Results:**

We found that 35 miRNAs and 301 genes were significantly differentially expressed. Functional enrichment analysis showed that upregulated genes were significantly enriched in extracellular matrix (ECM) organization, while downregulated genes were mainly involved in cell death and apoptotic process. Finally, we concluded the chemical-gene affected network, miRNA-mRNA interacted networks, and significant pathway network.

**Conclusion:**

We identified lists of differentially expressed miRNAs and genes and their possible interaction in pterygium. The networks indicated that ECM breakdown and EMT might be two major pathophysiological mechanisms and showed the potential significance of PI3K-Akt signalling pathway. MiR-29b-3p and collagen family (COL4A1 and COL3A1) might be new treatment target in pterygium.

## 1. Introduction

Pterygium is a common ocular surface disease, which is characterized by proliferation, inflammatory infiltrates, fibrosis, angiogenesis, and ECM breakdown in conjunctiva and progressively invaded the cornea [1, 2]. It is the result of an abnormal limbus basal epithelial stem cell that moves onto Bowman's layer and brings about the dissolution of this layer [3–5], affecting up to 200 million people globally and the prevalence in China has reached 108.65 million [6, 7]. Pterygium can impair vision, limit eye movements, and cause eye irritation, foreign body sensation, and dryness [8, 9]. Even more, if the pterygium grows over the entire corneal surface to block the visual axis and the patients will lose their vision [7, 10, 11]. However, so far, operation is the only effective way to deal with pterygium, but the recurrence is common. Accordingly, it is crucial to investigate the molecular mechanisms involved in the proliferation, apoptosis, and fibrosis of pterygium for the improvement of therapeutic strategies.

MicroRNAs are a large class of small, regulatory, noncoding RNAs that negatively regulate protein-coding genes in the posttranscription level by binding to the target mRNAs' 3' untranslated region (3'UTR) [12–14]. Recent studies have shown a tightly association between miRNAs and the pathogenesis of pterygium [6, 15–19]. Nowadays, high-throughput platform has become a mainstream method to identify general RNA (including noncoding RNA) alteration [20, 21]. Because of bioinformatics' information integration ability, it is conducive to get new direction effectively.

In this study, we pooled the expression profiling of differentially expressed miRNAs (DEMs) from published studies [6, 15–17] and analyzed differentially expressed genes (DEGs) with the GEO dataset (http://www.ncbi.nlm.nih.gov/geo/), a repository, which archives and distributes freely microarray, next-generation sequencing (NGS), and other forms of high-throughput functional genomic data [22, 23]. Then, functional enrichment and pathway network analysis were applied to identify the DEGs using DAVID (http://david.abcc.ncifcrf.gov/). The protein-protein interaction (PPI) network and the chemical-gene interaction network were obtained from STRING (http://string-db.org/) and the comparative toxicogenomics database (CTD, www.ctdbase.org). MiRNAs' target genes were identified with miRDB (http://www.mirdb.org/) and TargetScan (http://www.targetscan.org), in order to demonstrate the key molecules as well as their potential mechanisms in pterygium.

## 2. Materials and Methods

### 2.1. Literature Searching and MicroRNA Data

We have searched the GEO and ArrayExpress databases for published microarray data of dysregulated miRNAs in pterygium, but there was no applicable result. Then we made a systemic search of PubMed, Embase, and Web of Science and found 4 studies, which involved the microarray assays about DEMs in pterygium. All of the pterygium tissues used in microarray are primary pterygium tissue. Three of them used normal unaffected conjunctiva as controls, while Lee used normal Tenon's capsule tissue as control, which in front adhered to the conjunctiva. To find the significant DEMs in progression of pterygium, we combined the 4 studies and drew up the unified standard to extract appropriate genes with P value < 0.05 and |log⁡2Fold  Change  (FC)| > 1. Finally, the pooled miRNAs were presented with Venn diagram, which was drew online with the Draw Venn Diagram website (http://bioinformatics.psb.ugent.be/webtools/Venn/) and used as the DEMs in the following analysis.

### 2.2. Microarray Data of mRNA Expression and Data Processing

We searched the GEO database for publicly available studies by the following key words “pterygium” (study keyword) and “Homo sapiens” (organism), and then the gene expression profile (GSE2513) was downloaded from the GEO database. This microarray data for GSE2513 included 8 primary pterygium tissue samples and 4 control unaffected conjunctiva tissue samples. And then, we used* R* to screen DEGs between pterygium and control conjunctiva. The adjusted* P*-value using Benjamini and Hochberg (BH) false discovery rate (FDR) method by default was applied to correct for the occurrence of false positive results. The cut-off criteria were an adjusted* P*-value < 0.25 and a |log⁡2FC| > log_2_⁡1.5 for DEGs. The volcano plot and the heat map of DEGs expression (232 upregulated genes and 69 downregulated genes) were set up using R programming language (*R*) and HemI_1.0_alpha software (http://hemi.biocuckoo.org/down.php) [24, 25], respectively.

### 2.3. Function Enrichment and Pathway Network Analysis

We use Gene Ontology (GO, http://geneontology.org/), a common bioinformatics tool for the inference of functional relationships between gene products, to predict biological processes, cellular components and molecular functions [26, 27]. And the Kyoto Encyclopedia of Genes and Genomes (KEGG, http://www.genome.jp/kegg/) was used to indicate the building blocks (genes and molecules) and the network information, including molecular wiring diagrams (interaction/ reaction networks) and hierarchical classifications (relation networks) [28]. The GO and KEGG analysis were performed using the DAVID database, which means a common bioinformatics database for annotation, visualization, and integrated discovery, providing tools for the functional interpretation of large lists of genes or proteins [29]. The cut-off criterion of the DEGs identified in GSE2513 was set as* P* < 0.05. Then, the functional enrichment and pathway analysis of DEGs were showed with histogram and bubble chart, and the bubble chart was performed by the imageGP website (www.ehbio.com/ImageGP).

### 2.4. Protein-Protein Interaction Network and Module Analysis

We used the STRING database, online common software, for providing a critical assessment and integration of protein-protein interactions (PPI) of DEGs, including direct (physical) as well as indirect (function) association [30, 31]. The DEGs applied by* R *were mapped to STRING to create a PPI network and the cut-off level was set up as a confidence score > 0.4. Then we used Cytoscape software (www.cytoscape.org), the most popular open-source software tools for visual exploration of biomedical networks composed of proteins, genes, and other types of interaction, to visualize the PPI network[32]. We chose the node degree of > 10 for screening hub genes as the filter criterion. The Molecular Complex Detection (MCODE) plugin was used to screen modules of hub genes in the PPI network with the degree cut-off = 2, haircut on, node score cut-off = 0.2, k-core = 2, and max depth = 100. Another plugin in Cytoscape, cytoHubba, was used to calculate degrees of each protein node. We chose the top 20 genes as hub genes and analyzed the function enrichment and pathways with DAVID database.

### 2.5. Small Molecule Target Network Analysis

The comparative toxicogenomics database (CTD) is a public resource, which contains associations between chemicals, gene products and diseases [33]. Chemicals can change gene expression from the level of transcription, and the expression of gene in turn can regulate the reactivity of chemicals by affecting localization, binding cleavage, and so on. We appointed the disease as pterygium and identified the chemical small molecules associated with the DEGs. The chemical-gene interaction network was constructed with Cytoscape.

### 2.6. Construction of a miRNA-mRNA Regulatory Network

We identified miRNAs and mRNAs by screening the miRBase (http://www.mirbase.org) and Ensembl database (http://www.ensembl.org/index.html, version 94). MiRBase provided a wide-range of information on published miRNAs, including their sequences and deep sequencing expression data [34], while Ensembl provide genome interpretation [35]. In this study, miRNAs and mRNAs that were not included in the database were excluded and all the included miRNAs and mRNAs used official name. To further investigate the functional roles of the miRNAs, the putative target genes of the miRNAs were predicted by two databases, TargetScan and miRDB, which were most common in use for miRNAs target prediction and functional annotations [36, 37]. And a small portion of the predicted genes were retained, which is the intersection of target genes obtained from the two prediction databases. Those genes that expressed the opposite of their miRNA counterparts were further selected, to predict the potentially significant pathway with the DAVID database, and performed another network by using Cytoscape.

## 3. Results

### 3.1. DEMs in Pterygium

**Figure 1:**
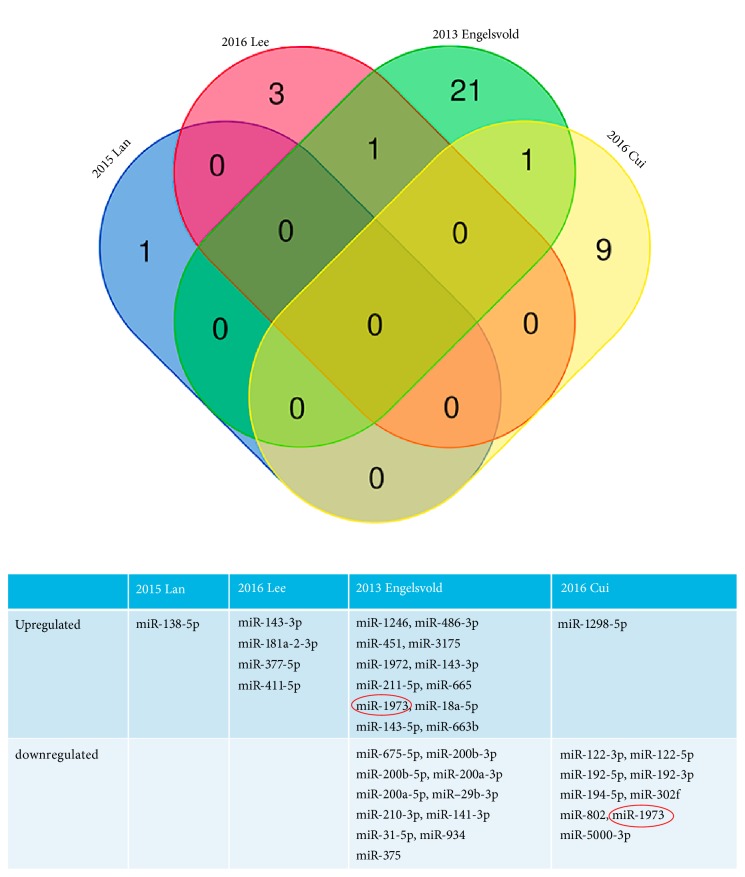
Analysis of the microRNA microarray results from 4 publications to explore the differentially expressed miRNA. With the criteria of* P* value <0.05 and |log⁡2FC| > 1, there were 17 upregulated miRNAs and 20 downregulated miRNAs.

Pooling the results of these 4 researches, there were 36 differentially expressed miRNAs that met the criteria of* P* value < 0.05 and |log⁡2FC| > 1 [6, 15–17], all of these DEMs' related information was shown in Table 1, and the pooling results were shown with Venn diagram. Among the 36 DEMs, miR-143-3p appeared twice and showed obvious high-expression in pterygium, while miR-1973 was excluded though appeared also twice but paradoxically express. The final expression profile of miRNAs in pterygium was shown in Figure 1. Among these DEMs, 16 miRNAs were upregulated, while 19 miRNAs were downregulated.

### 3.2. DEGs in Pterygium

**Figure 2:**
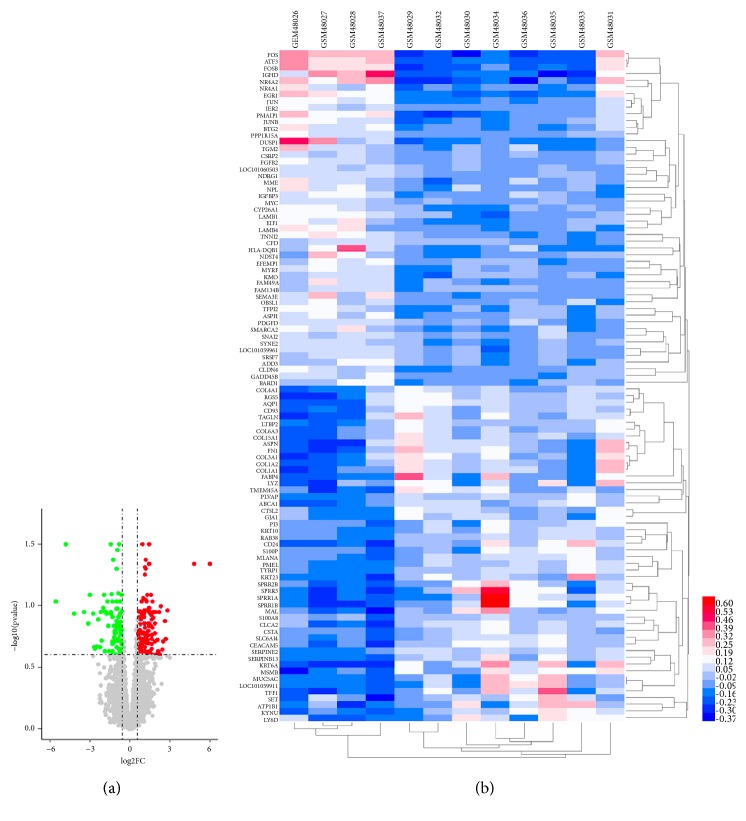
Differentially expressed genes obtained from GES2513. (a) Volcano plot. There were 232 upregulated genes and 69 downregulated genes up to the standard and among these differentially expressed genes, about 36% downregulated genes involved in apoptosis, such as ATF3, NR4A2, NR4A1, EGR1, and JUN (in the top 10 downregulated genes), and over half of upregulated genes were related to extracellular matrix (ECM), including collagen family (COL1A2, COL3A1, COL4A1, COL4A2, COL6A1, COL6A3, and COL15A1) and matrix metalloproteinases family (MMP2 and MMP7). (b) Heat map of top 50 downregulated genes and top 50 upregulated genes. The 4 GEO samples (GSM) in the left columns were collected from normal conjunctiva tissues of cataract patients, while the 8 GSMs in the right columns were taken from lesional tissues of patients with pterygium.

A total of 301 DEGs expressed in pterygium were extracted from the GSE2513 datasets with the criteria of* P* value < 0.25 and |log⁡2FC| > log_2_⁡1.5, including 232 upregulated genes and 69 downregulated genes in comparison to conjunctival control tissues. The volcano plot and heat map of DEGs expression were shown in Figure 2. Heat map only showed the top 50 downregulated and top 50 upregulated genes of all DEGs.

### 3.3. Functional Enrichment and Pathway Network Analysis

**Figure 3:**
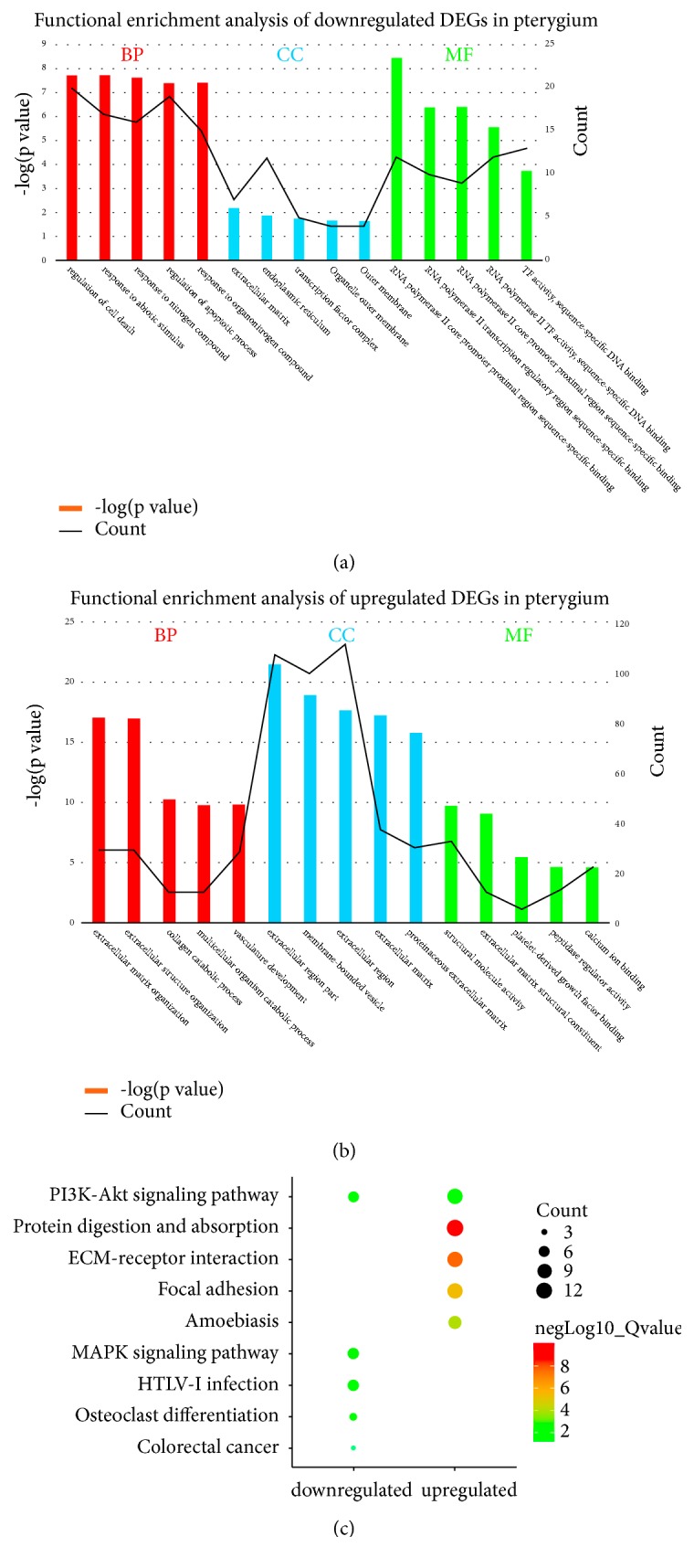
The functional enrichment and pathway analysis of DEGs in pterygium. (a) Functional enrichment analysis of downregulated DEGs. (b) Functional enrichment analysis of upregulated DEGs. (c) Bubble chart of pathway enrichment analysis of DEGs. BP represented biological processes, CC represented cellular components, and MF represented molecular functions.

The results demonstrated that a total of 886 GO terms or KEGG pathways were enriched (Supplementary 1), and the top 5 GO terms and KEGG pathways were selected based on the most significant. GO term enrichment analysis showed that upregulated DEGs were significantly enriched in ECM and structure organization, while downregulated DEGs were significantly enriched in regulation of cell death (Figures 3(a) and 3(b)). The upregulated DEGs were significantly enriched in five KEGG pathways, including ECM-receptor interaction, focal adhesion, and PI3K-Akt signalling pathway, while the downregulated DEGs were significantly enriched in MAPK signalling pathway, PI3K-Akt signalling pathway, osteoclast differentiation, and colorectal cancer (Figure 3(c)). And some of these GO terms and KEGG pathways were matched with the pathogenesis of pterygium reported, such as abnormal ECM, abnormal proliferation, and angiogenesis [2, 15, 38].

### 3.4. PPI Network Construction and Analysis of Modules

**Figure 4:**
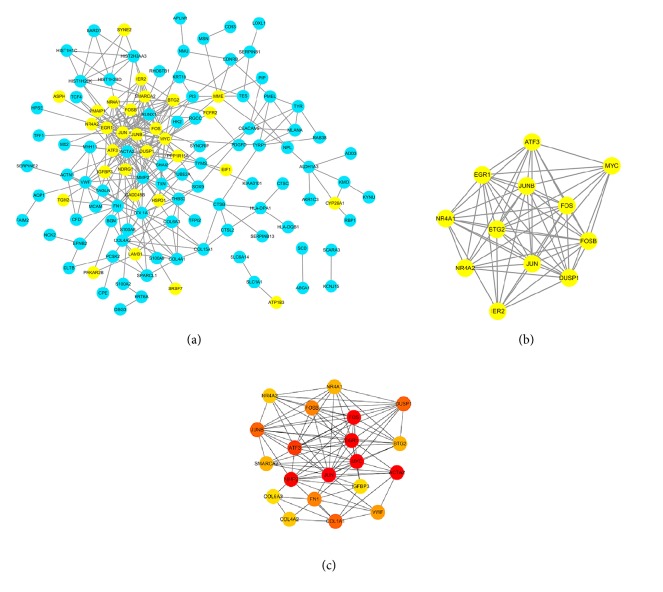
PPI network and hub genes. (a) The whole PPI network of DEGs. Blue nodes denoted upregulated genes, while yellow nodes denoted downregulated genes; the lines represented an interaction relationship between the nodes. (b) The most significant module selected from the PPI network, all genes in this module were downregulated. (c) The top 20 genes evaluated by connectivity degree in the PPI network, the color from red to yellow represented the connectivity degree from high to low.

The PPI network of DEGs was obtained by using the STRING database, including 118 nodes and 302 edges, and the nodes contain 40 downregulated genes and 78 upregulated genes (Figure 4(a)).

Furthermore, a significant module was generated by MCODE, including 12 nodes and 60 edges, and all genes in the module are downregulated (Figure 4(b)). The top 20 genes evaluated by connectivity degree in the PPI network were present by cytoHubba, and the result showed that JUN was the most significant gene with connectivity degree of 32, followed by MYC, ACTA2, EGR1, FOS, MMP2, ATF3, COL1A1, DUSP1, JUNB, FN1, FOSB, VWF, NR4A1, SMARCA2, BTG2, NR4A2, COL4A2, IGFBP3, and COL6A3 (Figure 4(c)). Functional enrichment and pathways analysis showed that ECM was at the center position and associated with multiple pathways (Table 2).

### 3.5. Target Network Analysis of Chemical Small Molecules

**Figure 5:**
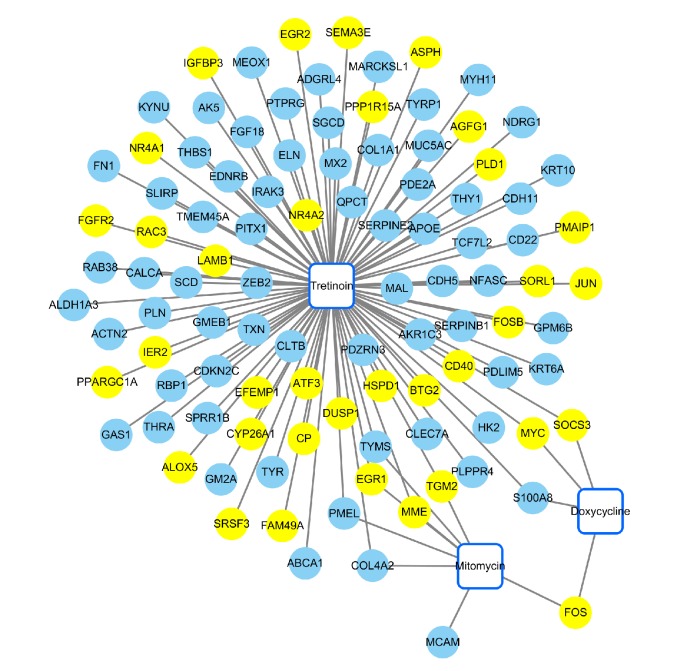
Chemical-DEGs interaction network. Blue squares represented the chemicals, blue nodes denoted upregulated genes, and yellow nodes denoted downregulated genes.

Following construction of the chemical-DEGs interaction network, 3 small molecules, which might be associated with pterygium, were identified, including tretinoin, mitomycin, and doxycycline (Figure 5).

### 3.6. miRNA-DEG Pairing and Relevant Pathways

**Figure 6:**
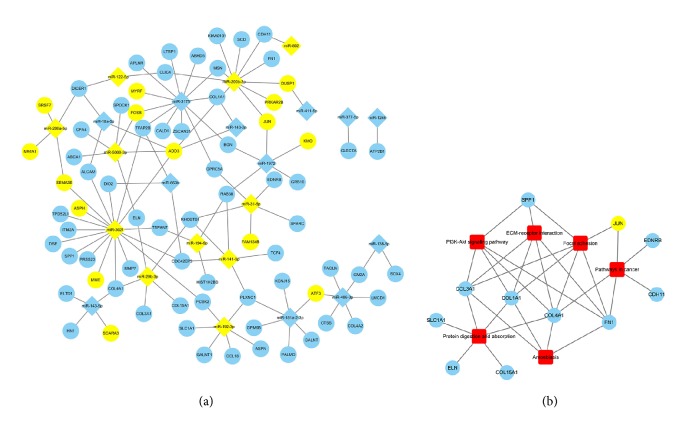
Networks between DEMs and target DEGs and significant DEGs and pathway. (a) The network of DEMs and their predicted target DEGs. Yellow circular node and diamond-shaped node denoted downregulated DEGs and DEMs, respectively, and blue circular node and diamond-shaped node denoted upregulated DEGs and DEMs, respectively. The lines represented a possibility of combination between the nodes. (b) The network of target DEGs and relevant pathways. Red round rectangle node denoted significant pathways, yellow and blue circular nodes denoted downregulated and upregulated DEGs, respectively, and the lines represented the DEGs which were involved in this pathway.

The TargetScan and miRDB database were used to predict the target genes of 35 DEMs identified from 4 published studies and we got intersection elements of the consistent target genes and DEGs from the GSE2513 (Figure 6(a)). Because the expression of target mRNA is almost opposite to that of miRNA, so we selected these genes with opposite expressed miRNAs and analyzed the potential and significant pathways with the DAVID database. We concluded a network of 6 significant pathways network using Cytoscape, including ECM-receptor interaction, protein digestion and absorption, focal adhesion, amoebiasis, PI3K-Akt signalling pathway, and pathways in cancer (Figure 6(b)). These results were coincident with the functional enrichment and pathway network analysis.

## 4. Discussion

Pterygium is a fibrovascular proliferative condition of the ocular surface, leading to ocular irritation, astigmatism, and even visual disturbance when it affects the visual axis [3–5, 39]. Because of the complicated pathological mechanism, pterygium bothers both of the patient and the surgeon because of its unsightly appearance and its tendency to recur [40].

Many miRNA have been reported to play key role in the occurrence and development of pterygium [6, 15–19, 41]. Both miR-215 and miR-221 exerted effects on fibroblast proliferation through its direct target genes functioning in cell cycling: downregulated miR-215 targeted Cdc25A and Mcm10 and promoted fibroblast proliferation, while upregulated miRNA-221 targets p27Kip1 gene, coding a cyclin-dependent kinase (CDK) inhibitor protein [6, 18]. However, miRNA-122 restrained pterygium epithelial cells apoptosis via targeting Bcl-w expression [17]. And miRNA-200 family, as the potential regulators of epithelial-mesenchymal transition (EMT), had an essential role in wound healing and tissue remodelling during pterygium occurrence [15, 42]. Some studies also showed that disordered miRNAs were speculated to be associated with the angiogenesis, induction of pluripotency genes, and repression of stem cell self-renewal [16, 19].

In order to find new nonsurgical treatments for pterygium, many studies involved molecular pathogenesis which were booming and from these researches, noncoding RNAs were a large group, suggested to be the novel molecular targets for treatment or therapeutic monitoring biomarkers [6, 15–18, 43, 44]. For instance, miR-216b's inhibition of apoptosis in fibroblasts in pterygium is opposite with the curative effect of hydroxycamptothecin, speculating that miR-216b inhibitor might be an effective therapeutic agents [45]. Although there were many potential applications in miRNAs-based treatment, challenges still remained, such as poor cellular absorptivity, action position, or target deviation and long-term safety in the body, which showed the significance in mechanisms study [46].

Our study attempted to predict the regulatory network between disordered miRNAs and genes in pterygium and potentially significant pathogenic mechanism. There were 232 upregulated genes and 69 downregulated genes up to the standard and among these differentially expressed genes, about 36% downregulated genes involved in apoptosis, such as ATF3, NR4A2, NR4A1, EGR1, and JUN (in the top 10 downregulated genes), and over half of upregulated genes were related to extracellular matrix (ECM), including collagen family (COL1A2, COL3A1, COL4A1, COL4A2, COL6A1, COL6A3, and COL15A1) and matrix metalloproteinases family (MMP2 and MMP7). 13.21% downregulated DEGs and 60.66% upregulated DEGs concentrated in extracellular region and matrix, including members of the matrix metalloproteinases family (MMP2 and MMP7) and fibrillary forming collagens (COL1A1 and COL1A2), which significantly occupied over half of all DEGs. Downregulated genes were significantly involved in cell death and apoptotic process, and both upregulated and downregulated genes showed PI3K-Akt signalling pathway's significance. Functional enrichment and pathway analysis of DEGs proved that pterygium might be the result of ECM disorder, apoptosis inhibition, and abnormal angiogenesis. The dominant one in the significant module was the AP-1 transcription factor family, including JUN, JUNB, FOS, and FOSB, which have been reported as regulators of cell proliferation, differentiation and apoptosis [47, 48]. Among the top 20 connective genes from PPI network, FN1, VWF, and IGFBP3 have been reported and expressed disorderly in pterygium [15, 49–51]. FN1 was involved in cell adhesion and migration processes, which has been found to enhance the EMT in pterygium, and IGFBP3 could control cell proliferation.

The two pathway analyses of hub genes and significant DEGs, which were the intersection of both DEMs' targets and DEGs, had something in common: ECM-receptor interaction, focal adhesion, pathways in cancer, and PI3K-Akt signalling pathway. These common grounds provided a comprehensive overview of the two major pathophysiological mechanisms of dysregulation in pterygium: EMT and ECM breakdown. It also showed that PI3K-Akt signalling pathway might be a significant pathway in pterygium, which played an important role in cell proliferation, differentiation, apoptosis, and even inflammation [2, 44, 52]. FN1 and the collagen family, including COL1A1, COL3A1, and COL4A1, were the components of ECM, involved in the fibrotic type of fibrosis and might be a group of significant genes in pterygium [15, 53, 54]. MiR-29b-3p and miR-200b-3p seemed to be the most significant miRNA, which target to more core DEGs. Since miR-200b-3p has been reported, miR-29b-3p needs experiments to show its differential expression in pterygium, and both could be considered in new treatment. All of these inferences and predictions gave us some new clues to find interacting mechanisms between miRNAs and target mRNAs and the pathophysiology of pterygium and provide theoretical support for follow-up studies on specific miRNAs as individualized medical treatment for prevention and treatment of pterygium.

Identification of small molecules with potential therapeutic efficacy for treatment was one of the aims of this study. Three chemicals were associated with pterygium, including tretinoin, doxycycline, and mitomycin. Tretinoin, an anti-inflammatory/angiogenesis agent, was testified to reduce IL-6, IL-8, and VEGF production in pterygium in study model, but has not been applied clinically [55]. Doxycycline has been a successful adjunctive treatment for pterygium, because of its significant inhibition of angiogenesis [56, 57]. Mitomycin C, considered in antirecurrent adjuvant therapy in some case to inhibit the cellular proliferation and migration, could be used in pterygium [58, 59], but because of the possibility of serious late complications, it always reserved for patients who had high probability of recurrence after excision of pterygium [60, 61]. However, their roles in the treatment of pterygium might have some limitations in application and require further investigation. Both chemicals and miRNAs could be new clinical treatment to reduce the necessity for surgical intervention and possibility of recurrence of pterygium.

There are some limitations in our study. We set the* P* value of DEGs from GSM2513 as 0.25 in order to get more genes for displaying network; on the other hand, there might be some genes, which were not expressed differentially in pterygium collected in the experiment. Many target genes predicted by databases were not negatively expressed with the DEMs, and these associations between DEGs and DEMs still need dual luciferase reporter assay and other experiments to validate.

## 5. Conclusion

Our study firstly explored the relationship between the DEGs and DEMs in pterygium. It indicated miR-29b-3p might be implicated in the development of pterygium and the collagen family, including COL3A1 and COL4A1 regulated by miR-29b-3p and associated with PI3K-Akt signalling pathway, might serve roles in the pathogenesis of pterygium. Furthermore, many DEGs were ECM proteins or associated with EMT indicating that ECM breakdown and EMT might be two of the most significant factors in pterygium formation. However, in vitro and in vivo studies are required to confirm the role these identified genes and pathways in the pathogenesis of pterygium.

## Figures and Tables

**Table 1:** Differentially expressed miRNAs in pterygium from 4 studies.

	Annotation	Fold change	*P* value	Microchip type
Lan 2015	miR-138-5p	3.0	0.019	Exiqon miRCURY LNA™ microRNA Array (4 pterygium tissue – 4 control tissue)

Lee 2016	miR-143-3p	2.4		GeneChip miRNA3.0 Array, Affymetrix (pterygium fibroblasts – control fibroblasts)
	miR-181a-2-3p	3.4		
	miR-377-5p	2.1		
	miR-411-5p	3.9		
	miR-145-3p	2.1		

Engelsvold 2013	miR-1246	4.5	0.001	GeneChip® miRNA2.0 Array, Affymetrix (8 pterygium tissue – 8 control tissue)
	miR-486-3p	4.4	0.004	
	miR-451	4.1	0.010	
	miR-3175	3.3	<0.001	
	miR-1972	3.0	<0.001	
	miR-143-3p	2.7	0.008	
	miR-211-5p	2.7	0.030	
	miR-665	2.3	0.010	
	miR-1973	2.2	0.040	
	miR-18a-5p	2.1	0.004	
	miR-143-5p	2.0	0.006	
	miR-663b	2.0	0.020	
	miR-675-5p	-2.0	0.005	
	miR-200b-3p	-2.1	0.002	
	miR-200b-5p	-2.3	<0.001	
	miR-200a-3p	-2.7	0.002	
	miR-200a-5p	-2.3	<0.001	
	miR-29b-3p	-2.3	0.005	
	miR-210-3p	-2.4	<0.001	
	miR-141-3p	-2.5	<0.001	
	miR-31-5p	-2.6	0.020	
	miR-934	-3.0	<0.001	
	miR-375	-3.7	0.030	

Cui 2016	miR-1298-5p	2.571	0.019	Exiqon miRCURY LNA™ microRNA Array (3 pterygium tissue – 3 control tissue)
	miR-122-3p	-14.7	0.005	
	miR-122-5p	-7.4	0.037	
	miR-192-3p	-3.3	0.006	
	miR-192-5p	-4.9	<0.001	
	miR-194-5p	-5.7	0.015	
	miR-302f	-2.8	0.006	
	miR-802	-4.1	0.039	
	miR-1973	-2.4	0.036	
	miR-5000-3p	-2.5	0.04	

**Table 2:** Functional and pathway enrichment analysis of hub genes.

Category	Term/Description	Count	*P* value
GOTERM_BP_FAT	GO:0010243~response to organonitrogen compound	12	1.35E-10
GOTERM_BP_FAT	GO:1901698~response to nitrogen compound	12	5.18E-10
GOTERM_BP_FAT	GO:0009725~response to hormone	11	7.24E-09
GOTERM_BP_FAT	GO:0071495~cellular response to endogenous stimulus	12	1.01E-08
GOTERM_BP_FAT	GO:0009719~response to endogenous stimulus	13	1.08E-08
GOTERM_CC_FAT	GO:0005578~proteinaceous extracellular matrix	6	2.39E-05
GOTERM_CC_FAT	GO:0031012~extracellular matrix	6	2.73E-04
GOTERM_CC_FAT	GO:0098644~complex of collagen trimers	3	3.21E-04
GOTERM_CC_FAT	GO:0044420~extracellular matrix component	4	4.15E-04
GOTERM_CC_FAT	GO:0005667~transcription factor complex	4	5.34E-03
GOTERM_MF_FAT	GO:0000982~transcription factor activity, RNA polymerase II core promoter proximal region sequence-specific binding	10	8.82E-11
GOTERM_MF_FAT	GO:0001228~transcriptional activator activity, RNA polymerase II transcription regulatory region sequence-specific binding	9	2.20E-09
GOTERM_MF_FAT	GO:0001077~transcriptional activator activity, RNA polymerase II core promoter proximal region sequence-specific binding	8	7.56E-09
GOTERM_MF_FAT	GO:0000981~RNA polymerase II transcription factor activity, sequence-specific DNA binding	10	2.85E-08
GOTERM_MF_FAT	GO:0044212~transcription regulatory region DNA binding	9	3.61E-06
KEGG_PATHWAY	hsa04512:ECM-receptor interaction	5	6.38E-05
KEGG_PATHWAY	hsa04510:Focal adhesion	6	1.43E-04
KEGG_PATHWAY	hsa04151:PI3K-Akt signaling pathway	7	1.69E-04
KEGG_PATHWAY	hsa05200:Pathways in cancer	6	2.74E-03
KEGG_PATHWAY	hsa04010:MAPK signaling pathway	5	3.64E-03

*∗* If there were more than 5 terms enriched in this category, the top 5 terms were selected according to *P* value.

## Data Availability

The microarray data used to support the findings of this study have been deposited in the GEO database (GSE2513).

## Supplementary Materials

Supplementary Materials It is the complete list of original data from DAVID of differentially expressed genes (DEGs). There are 8 sheets, including downregulated DEGs-BP, downregulated DEGs-CC, downregulated DEGs-MF, downregulated DEGs-KEGG, upregulated DEGs-BP, upregulated DEGs-CC, upregulated DEGs-MF, and upregulated DEGs-KEGG, covering the biological process, cell component, molecular function, and pathway analysis of downregulated and upregulated DEGs, respectively (xlsx, 177kb).
